# Anti-Hyperuricemic and Nephroprotective Effects of Dihydroberberine in Potassium Oxonate- and Hypoxanthine-Induced Hyperuricemic Mice

**DOI:** 10.3389/fphar.2021.645879

**Published:** 2021-04-20

**Authors:** Lieqiang Xu, Guoshu Lin, Qiuxia Yu, Qiaoping Li, Liting Mai, Juanjuan Cheng, Jianhui Xie, Yuhong Liu, Ziren Su, Yucui Li

**Affiliations:** ^1^ School of Pharmaceutical Sciences, Guangzhou University of Chinese Medicine, Guangzhou, China; ^2^ State Key Laboratory of Dampness Syndrome of Chinese Medicine, The Second Affiliated Hospital of Guangzhou University of Chinese Medicine, Guangzhou, China; ^3^ Guangdong Provincial Key Laboratory of Clinical Research on Traditional Chinese Medicine Syndrome, Guangzhou, China

**Keywords:** hyperuricemia, inflammation, xanthine oxidase, NLRP3 inflammasome, urate transporters, dihydroberberine

## Abstract

Phellodendri Chinese Cortex has long been used to treat hyperuricemia and gout. Berberine (BBR), its characteristic ingredient, has also been shown to be effective in alleviating monosodium urate crystals-triggered gout inflammation *in vitro* and *in vivo*. Dihydroberberine (DHB) is a hydrogenated derivative of BBR that showed improved *in vivo* efficacy on many metabolic disorders. However, its anti-hyperuricemia effect remains underexplored. In the present work, the hypouricemic and renoprotective effects of DHB on hyperuricemic mice were investigated. The hyperuricemic mice model was induced by intraperitoneal injection of potassium oxonate (PO, 300 mg/kg) combined with intragastric administration of hypoxanthine (HX, 300 mg/kg) for 7 days. Different dosages of DHB (25, 50 mg/kg), BBR (50 mg/kg) or febuxostat (Feb, 5 mg/kg) were orally given to mice 1 h after modeling. The molecular docking results showed that DHB effectively inhibited xanthine oxidase (XOD) by binding with its active site. *In vitro*, DHB exhibited significant XOD inhibitory activity (IC_50_ value, 34.37 μM). The *in vivo* results showed that DHB had obvious hypouricemic and renoprotective effects in hyperuricemic mice. It could not only lower the uric acid and XOD levels in serum, but also suppress the activities of XOD and adenosine deaminase (ADA) in the liver. Furthermore, DHB noticeably down-regulated the renal mRNA and protein expression of XOD. Besides, DHB remarkably and dose-dependently ameliorated renal damage, as evidenced by considerably reducing serum creatinine and blood urea nitrogen (BUN) levels, inflammatory cytokine (TNF-α, IL-1β, IL-6 and IL-18) levels and restoring kidney histological deteriorations. Further mechanistic investigation showed that DHB distinctly down-regulated renal mRNA and protein levels of URAT1, GLUT9, NOD-like receptor 3 (NLRP3), apoptosis-associated speck-like (ASC), caspase-1 and IL-1β. Our study revealed that DHB had outstanding hypouricemic and renoprotective effects via suppressing XOD, URAT1, GLUT9 and NLRP3 inflammasome activation in the kidney.

## Introduction

Hyperuricemia (HUA), a common extensive metabolic disease, is most commonly caused by excessive production and/or inadequate excretion of uric acid (UA) (Liang et al., 2019). It is well known that hyperuricemia is a potential risk factor for gout and has also become an independent risk of multiple metabolic disorders including diabetes (Lv et al., 2013), hypertension (De Becker et al., 2019), atherosclerosis (Liu Z. et al., 2016), and renal disease (Rodenbach et al., 2015). In recent years, the prevalence of hyperuricemia and gout has risen globally as a result of over-consumption of purine-rich foods. The US National Health and Nutrition Examination Survey (NHANES) study conducted in 2015–2016 showed that the prevalence of hyperuricemia among US men and women was 20.2 and 20.0%, respectively (Chen-Xu et al., 2019). And a meta-analysis indicated the prevalence of hyperuricemia was 17.4% (95% CI: 15.8–19.1%) in mainland China (Huang et al., 2020). Hence, hyperuricemia has emerged as a serious public health issue that seriously affects the life of patients and leads to the serious social financial burden, which deserves global concern.

The general prevention and treatment strategy against hyperuricemia is lowering the levels of serum uric acid (SUA), which entails reducing urate formation and/or enhancing the urate excretion. Xanthine oxidase (XOD), the key rate-limiting enzyme in UA metabolism, can oxidize hypoxanthine into xanthine and subsequently oxidize xanthine into UA (Zhang et al., 2018). Therefore, XOD can be regarded as a vital target for reducing UA production. Allopurinol and febuxostat, the two most commonly used drugs in clinics for the treatment of hyperuricemia, can lower the SUA levels through targeting XOD (Becker et al., 2005). Nevertheless, both febuxostat and allopurinol are demonstrated to produce side effects such as renal and gastrointestinal toxicity, liver and kidney damage, and myelosuppression, which compromise their clinical applications (Edwards, 2009; Strilchuk et al., 2019). The kidney acts a pivotal part in urate metabolism mainly through four processes: glomerular filtration, tubular reabsorption, secretion, and post-secretory reabsorption. Growing evidence supports the notion that urate reabsorption and secretion are controlled by urate transporters-related proteins. Urate transporter 1 (URAT1) and glucose transporter 9 (GLUT9) have been reported to mainly facilitate urate reabsorption (Wang et al., 2016). The dysregulated expressions of these urate transporters cause a reduction of urate excretion and result in hyperuricemia. Uricosuric agents including benzbromarone (URAT1 inhibitor) are commonly employed in clinical practice to treat hyperuricemia. However, these medicines also have occasional side effects, which create a stumbling block for their wide application (Strilchuk et al., 2019). Therefore, it is highly warranted to identify alternative promising candidate for the treatment of hyperuricemia.

The NOD-like receptor 3 (NLRP3) mediated inflammation is closely associated with the pathogenesis of various forms of renal disorders and their complications, including hyperuricemia (Hongyan et al., 2016). UA exerts potent pro-inflammatory effect on driving the activation of NLRP3 inflammasome. After activation, NLRP3 is oligomerized and interacts with ASC, which in return recruits effector protein caspase-1 forming the inflammasome complex. The activation of caspase-1 subsequently leads to maturation and secretion of interleukin (IL)-1β and IL-18, which cause severe inflammatory response (Braga et al., 2017). Therefore, inhibition of the NLRP3 inflammasome is a feasible strategy to alleviate kidney inflammation and damage in hyperuricemia.

Phellodendri Chinese Cortex (PC), also known as Huangbo in Chinese, is the dried trunk bark of *Phellodendron chinense* Schneid. It has long been used to treat various inflammation-related diseases (Kim et al., 2011). Owing to its excellent heat-clearing and dampness-eliminating effect in traditional Chinese medicine (TCM), PC is applied as a monarch herb (the key ingredient) in many famous TCM prescriptions, such as *Er-Miao-Wan*, *San-Miao-Wan*, and *Si-Miao-Wan*. Those prescriptions have long been used to treat gout. Modern pharmacological studies have proved their potent XOD-inhibitory activity and SUA-lowering effect (Kong et al., 2004; Wang et al., 2010). Berberine (BBR), the main component of PC, is effective in alleviating monosodium urate (MSU) crystals-triggered gout inflammation *in vitro* and *in vivo*, and the beneficial effect is partly attributed to the inactivation of NLRP3 inflammasome (Liu Y.-F. et al., 2016; Dinesh and Rasool, 2017). Besides, BBR exerts nephroprotective effects in experimental renal injury caused by cisplatin (Domitrovic et al., 2013), lead (Hasanein and Riahi, 2018), methotrexate (Hassanein et al., 2019), and mercury (Othman et al., 2014). However, its clinical use is limited due to its poor oral bioavailability and intestinal absorption. Dihydroberberine (DHB), a hydrogenated derivative of BBR, possesses favorable safety profile (LD_50_ value, 503.80 mg/kg) (Li, 2019). Our previous work has found that DHB possesses pronounced anti-inflammatory properties *in vitro* and *in vivo* (Li et al., 2019; Tan et al., 2019). Furthermore, previous research has indicated that the intestinal absorption rate of DHB is 5-fold higher than that of BBR (Feng et al., 2015). Besides, several studies have revealed that DHB has notable therapeutic efficacy on experimental diabetes (Turner et al., 2008), hyperlipidemia (Wei et al., 2014; Liu et al., 2015), and atherosclerosis (Chen et al., 2014), etc.

Based on the above-mentioned findings, DHB may have the potential to be further developed as a promising drug candidate for the treatment of hyperuricemia. However, to our knowledge, the hypouricemic and renoprotective effects of DHB on experimental hyperuricemia animal models remain obscure. Therefore, this study was designed to explore the potential hypouricemic and nephroprotective effects of DHB using the PO/HX-induced hyperuricemic mice model.

## Materials and Methods

### Chemicals and Materials

**FIGURE 1:**
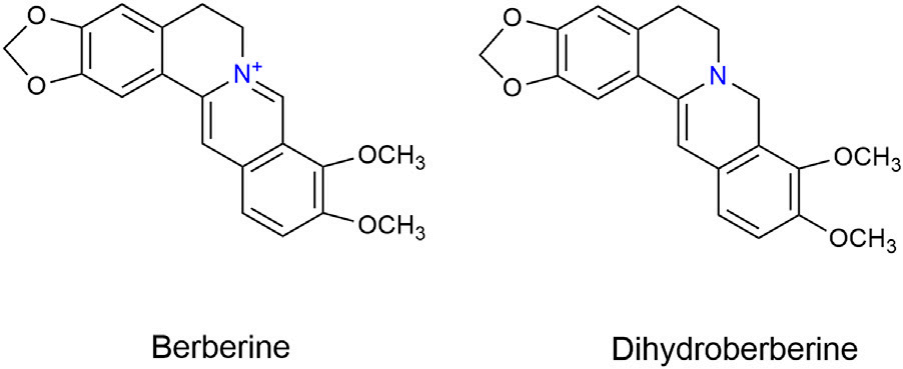
Chemical structures of BBR and DHB.

Xanthine oxidase (XOD, EC 1.1.3.22) from cow milk (1 unit/mg protein), potassium oxonate (PO) and hypoxanthine (HX) were purchased from Sigma-Aldrich Chemical Co. (St. Louis, MO, United States). Xanthine was obtained from Dalian Meilun Biotech Co., Ltd. (Dalian, China). Allopurinol was obtained from Hebei Bailing Weichao Fine Materials Co., Ltd. (Langfang, China). Febuxostat (Feb) was bought from Shanghai yuanye Bio-Technology Co., Ltd. (Shanghai, China). BBR and DHB (Figure 1) were obtained from Chengdu HerbPurity CO., Ltd. (Chengdu, China). Assay kits for determination of SUA, blood urea nitrogen (BUN), creatinine, XOD and adenosine deaminase (ADA) were obtained from Nanjing Jiancheng Biotechnology Institute (Nanjing, China). Enzyme-linked immunoabsorbent commercial cytokine assay kits for murine TNF-α, IL-1β, IL-6 and IL-18 were purchased from Shanghai Chengshao Biotech Co., Ltd. (Shanghai, China). Mouse primers for ASC, caspase-1, NLRP3, IL-1β, URAT1, GLUT9 and XOD were purchased from Shanghai Sangon Biological Engineering Co., Ltd (Shanghai, China). Antibodies against NLRP3, ASC, IL-1β, and caspase-1 were obtained from Affinity Biosciences (Cincinnati, OH, United States). Anti-URAT1. anti-GLUT9 and anti-GAPDH were purchased from Proteintech Group, Inc. (Wuhan, China), and anti-XOD was purchased from Abcam (Cambridge, MA, United States).

### Animals

Seventy SPF male KM mice (4 weeks, 24 ± 2 g) were purchased from Laboratory Animal Center of Guangzhou University of Chinese Medicine (GZUCM, Guangzhou, China). In order to acclimate to a new environment, mice were maintained in an SPF facility with a 12/12 h light/dark photoperiod (25 ± 1°C, 60 ± 10% humidity) in the animal house, and fed with laboratory regular diet and sterilized water *ad libitum* for 1 week before performing any procedure. All animal procedures were conducted in compliance with the guidelines for Care and Use of Laboratory Animals of GZUCM. Ethical approval for the animal experiments was granted by the Animal Ethics committee of GZUCM (Permit ID: 20190919002).

### Experimental Protocols

In this study, the hyperuricemic mouse model was constructed by co-treatment with PO and HX according to the previously published methods (Yong et al., 2016; Yong et al., 2017). All mice were randomly assigned to 6 different groups (10 mice per group): control (NC), hyperuricemic (HUA), febuxostat (Feb, 5 mg/kg), BBR (100 mg/kg), and DHB (25 and 50 mg/kg). Feb, PO, HX, BBR, and DHB were suspended in 0.5% sodium carboxymethylcellulose (CMC-Na) freshly. The hyperuricemia mouse model was generated via intraperitoneal (i.p.) administration of 300 mg/kg PO and oral gavage (p.o.) of 300 mg/kg HX 1 h before the drug interventions for all groups except NC group (Chau et al., 2019); the NC group were treated with the same volume of 0.9% physiological saline solution (i.p.) and 0.5% CMC-Na (p.o.). One hour after modeling, mice in Feb group were orally given febuxostat (5 mg/kg) and mice in drug groups orally received BBR (50 mg/kg) or DHB (25 and 50 mg/kg) once daily for 1 week, respectively.

After fasted overnight, all animals were anesthetized 1 h after the drug supplementation on day 7. The blood was obtained from abdominal aorta and centrifuged at 3,000 rpm for 10 min at 4°C. Serum was separated and stored in aliquots at -80°C for biochemical assays. After blood collection, liver and kidney were removed, rinsed, and weighed rapidly. Liver tissue was cut into pieces, mixed with 0.9% physiological saline solution of approximately 9-fold volume of the sample weight, and homogenized in a homogenizer on ice. The homogenate was centrifuged at 3,000 rpm at 4°C for 10 min and supernatants harvested were frozen until XOD and ADA activity assay. Kidney tissues were divided into three parts, for histopathological evaluation, ELISA test, RT-PCR and Western blot analysis, respectively.

### XOD Inhibition Assay

The *in vitro* XOD inhibitory capacity of DHB was performed spectrophotometrically at 290 nm according to a previously published method with minor modifications (Rahmi et al., 2020). Firstly, 50 μl of test sample (dissolved in DMSO) and 7 μl of XO enzyme solution (0.4 units/mL in PBS with pH at 7.2) were mixed and incubated in wells of the 96-well microtitration plate at 37°C for 15 min. Then, 66 μl of xanthine substrate (500 μmol/L in PBS with pH at 7.2) was added into the mixture. After 30 min of incubation at 37°C, 50 μl of 1% HCl was added, and the absorbance was determined at 290 nm with a microplate reader. Allopurinol served as a positive control. A blank of each sample was prepared replacing by XOD with PBS. Every reaction was repeated three times. XOD inhibitory activity was calculated as $\left[1-\frac{\left(S-S_{0}\right)}{\left(B-B_{0}\right)}\right]\times 100$, where S and S_0_ are the activities of the sample with and without XOD, respectively, B is the enzyme activity without sample, and B_0_ is the control of B without sample and XOD.

### Biochemical Assay

Serum levels of XOD, UA, creatinine, and BUN, as well as XOD and ADA in the supernatants of liver homogenates were measured with appropriate standard kits according to the manufacturers’ instructions. To evaluate the general toxicity, body weight was recorded daily, and organ weight and organ index were measured at the end of experiment. For liver, protein content was measured using a BCA protein assay kit by comparison with a known concentration of bovine serum albumin.

### Histological Assay

Kidney tissues were fixed in 4% paraformaldehyde for more than 24 h and subsequently routinely embedded in paraffin, sectioned into 3 µm thick sections and stained with H&E staining. Then the treated slices were photographed under Olympus BX53 optical microscopy (Olympus, Tokyo, Japan) at 100, 200, and ×400 magnification, respectively.

### Gene Expression

Liver and kidney were processed for total RNA extraction using Trizol reagent (Invitrogen) in accordance with the standard protocol. Then reverse transcription of RNA sample to cDNA was conducted by the PrimeScript RT reagent kit (Takara, Tokyo, Japan). The mRNA expression levels of URAT1, GLUT9, ASC, caspase-1, NLRP3, IL-1β and XOD were measured using SYBR Green Master Mix Kit (Vazyme Biotech Co., Ltd., China) on a CFX System (Bio-Rad). All primer sequences are summarized in Table 1. The relative mRNA expression and fold change of target genes were based on the 2^−∆∆Ct^ method and normalized against β-actin, which served as the internal reference.

**TABLE 1:** Sequences of the primers for real time RT-PCR analysis.

Description	Genebank	Sequence of primers (5′–3′)	Product size (bp)	Tm (◦C)
XOD	NM 011723	ATG​ACG​AGG​ACA​ACG​GTA​GAT	185	55.3
		TCA​TAC​TTG​GAG​ATC​ATC​ACG​GT		55.0
URAT1	NM 009203	CGT​GGG​ACC​TGG​TAT​GTA​ACT	112	56.5
		CCA​AAC​CTA​TCT​GAG​GCA​TGG		55.8
GLUT9	NM_001,102,414	TTG​CTT​TAG​CTT​CCC​TGA​TGT​G	154	55.1
		GAG​AGG​TTG​TAC​CCG​TAG​AGG		56.9
NLRP3	NM_001,359,638	ATT​ACC​CGC​CCG​AGA​AAG​G	141	57.7
		TCG​CAG​CAA​AGA​TCC​ACA​CAG		58.1
ASC	NM_023258	ACA​ATG​ACT​GTG​CTT​AGA​GAC​A	81	53.2
		CAC​AGC​TCC​AGA​CTC​TTC​TTT​A		53.8
Caspase-1	NM_009807	AGA​GGA​TTT​CTT​AAC​GGA​TGC​A	183	53.3
		TCA​CAA​GAC​CAG​GCA​TAT​TCT​T		53.3
IL-1β	NM_008361	GCA​ACT​GTT​CCT​GAA​CTC​AAC​T	89	55.2
		ATC​TTT​TGG​GGT​CCG​TCA​ACT		56.4
β-actin	NM_007393	GTGCTATGTTGCTCTAGACTTCGATGCCACAGGATTCCATACC	174	55.354.7

### Western Blot Analysis

Liver and kidney were weighed and homogenized in 10 equivalent volumes of ice-cold RIPA lysis buffer, centrifuged at 12,000 rpm for 10 min at 4°C to obtain total proteins, followed by determining concentration by BCA method. Then the total proteins were mixed with denaturing loading buffer, and boiled for 10 min. Adjusted aliquots of mixtures were subjected to SDS-PAGE gel, and then the fully separated proteins were transferred to a PVDF membrane in the presence of a blocking solution (TBS-T containing 5% skim milk) for 1 h. Subsequently, the membranes were incubated with primary antibodies: anti-URAT1 (1: 1,000), anti-GLUT9 (1: 1,000), anti-NLRP3 (1: 2,000), anti-ASC (1: 1000), anti-caspase-1 (1: 1,000), anti-IL-1β (1: 1,000) and anti-GAPDH (1: 20,000) at 4°C overnight, followed by secondary HRP-conjugated goat anti-rabbit IgG (Immunoglobulin G, 1:4,000) antibody for 60 min. Eventually, the membranes were mixed with ECL and photographed under transmitted ultraviolet light. The protein bands were analyzed by ImageJ software (NIH, Bethesda, MD, United States) and normalized to the expression of GAPDH.

### Molecular Docking

A molecular docking study was conducted to explore the feasible interaction model of DHB against XOD using the AutoDock Vina software. XOD crystal structure (PDBID: 1FIQ, resolution: 2.50 Å) was downloaded from the RCSB PDB databases (http://www.rcsb.org/), which was set as receptor. The 3D structure of Dihydroberberine (PubChem CID: 10217) was retrieved from the PubChem database (https://pubchem.ncbi.nlm.nih.gov/), and thereafter exploited as ligands. The active pocket was defined as a sphere of 10 Å in diameter, which located 2-hydroxybenzoic acid as the center and then removed. Top ranked poses with the lowest energy score were considered privileged binding model, which were selected for further study. And the docking results were visualized by Pymol software (version 1.6, http://www.pymol.org).

### Statistical Analysis

The SPSS 19.0 software was used for the statistical analysis (Chicago, IL, United States). And results are presented as means ± SD. For multiple treatment groups comparisons, One-way ANOVA and LSD test were applied. *p*-values < 0.05 were considered to being significant.

## Results

### Effect of DHB Treatment on Body Weight and Organ Coefficient

The changes in body weight, organ weight (liver and kidney) and organ coefficients (liver and kidney) are showed in Table 2. During the experiment, no death or significant difference in body weight were observed between any of the groups. Besides, there was no noticeable difference in liver weight and liver index between groups. However, hyperuricemia significantly increased the kidney weight (0.385 g) and kidney index (1.606%) in comparison to the NC group (kidney weight: 0.274 g, *p* < 0.01; kidney index: 1.055%, *p* < 0.01). However, DHB and BBR administration significantly inhibited the elevated levels of the above parameters.

**TABLE 2:** Effect of DHB on body weight, organ weight and organ index.

Group	Body weight (g)	Organ weight (g)		Organ index (%)	
		Liver	Kidney	Liver	Kidney
NC	25.05 ± 1.61	0.95 ± 0.12	0.27 ± 0.03	3.77 ± 0.38	1.06 ± 0.08
HUA	25.71 ± 1.44	1.01 ± 0.12	0.39 ± 0.03^##^	3.92 ± 0.36	1.61 ± 0.14^##^
Feb (5)	25.61 ± 1.66	1.01 ± 0.13	0.30 ± 0.02**	4.03 ± 0.34	1.19 ± 0.08**
DHB (25)	25.47 ± 2.22	0.96 ± 0.12	0.32 ± 0.03**	3.78 ± 0.32	1.35 ± 0.17**
DHB (50)	26.03 ± 2.47	1.02 ± 0.14	0.31 ± 0.04**	3.97 ± 0.34	1.16 ± 0.11**
BBR (50)	24.57 ± 2.00	0.95 ± 0.18	0.29 ± 0.06**	3.71 ± 0.36	1.14 ± 0.13**

The results are expressed as mean ± SD (n = 10).

^*#*^ *p* < 0.05, ^*##*^ *p* < 0.01, compared with NC group; ^*^ *p* < 0.05; ^**^ *p* < 0.01, compared with HUA group.

### Effect of DHB Treatment on UA, Creatinine, and BUN

**FIGURE 2:**
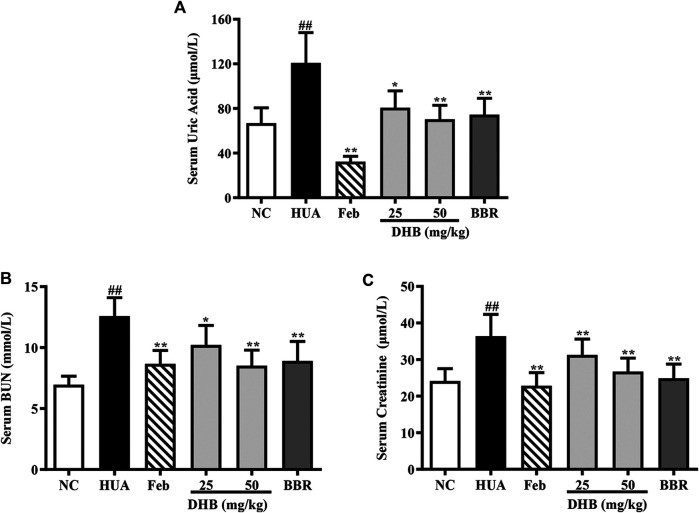
Effect of DHB on serum UA and serum markers of kidney damage in PO/HX-induced hyperuricemic mice. **(A)** The serum level of UA. **(B)** The serum level of BUN. **(C)** The serum level of creatinine. The results are expressed as mean ± SD (n = 10). ^#^ *p* < 0.05, ^##^ *p* < 0.01, compared with NC 616 group; ^*^ *p* < 0.05; **p < 0.01, compared with HUA group.

SUA level, which has been widely accepted as a reliable indicator for hyperuricemia, was determined. As shown in Figure 2A, PO/HX administration caused a noticeable elevation by 80.80% in SUA concentration as compared to the NC group (*p* < 0.01), indicating the successful construction of HUA model. After DHB treatment for 7 days, SUA levels significantly decreased from 120.91 μmol/L (HUA group) to 80.68 μmol/L (25 mg/kg, *p* < 0.01) and 70.27 μmol/L (50 mg/kg, *p* < 0.01), respectively, which was close to the NC group (66.87 μmol/L). Oral treatment with Feb (5 mg/kg) elicited a notable decrease in SUA to 32.38 μmol/L (*p* < 0.01), which was significantly lower than that of the NC group (*p* < 0.01). BBR (50 mg/kg) also significantly lowered the SUA to 74.53 μmol/L without any significant difference in comparison with the NC group.

The serum levels of creatinine and BUN, two closely related parameters indicative of renal dysfunction, were detected, and the results are displayed in Figures 2B,C. As expected, the serum creatinine and BUN values of HUA group noticeably elevated around 50.84 and 80.92% respectively as compared to those of the NC group (all *p* < 0.01), indicative of serious impairment of kidney injury. While Feb and BBR effectively reversed PO/HX-induced increase of BUN (*p* < 0.01) and creatinine (*p* < 0.01) levels. Furthermore, DHB at different doses significantly reduced BUN level by 18.79% (25 mg/kg, *p* < 0.01) and 32.31% (50 mg/kg, *p* < 0.01), respectively, and creatinine level by 14.08% (25 mg/kg, *p* < 0.01) and 26.58% (50 mg/kg, *p* < 0.01), suggestive of some protective effect on kidney function.

### Effect of DHB Treatment on Renal Histopathological Features

**FIGURE 3:**
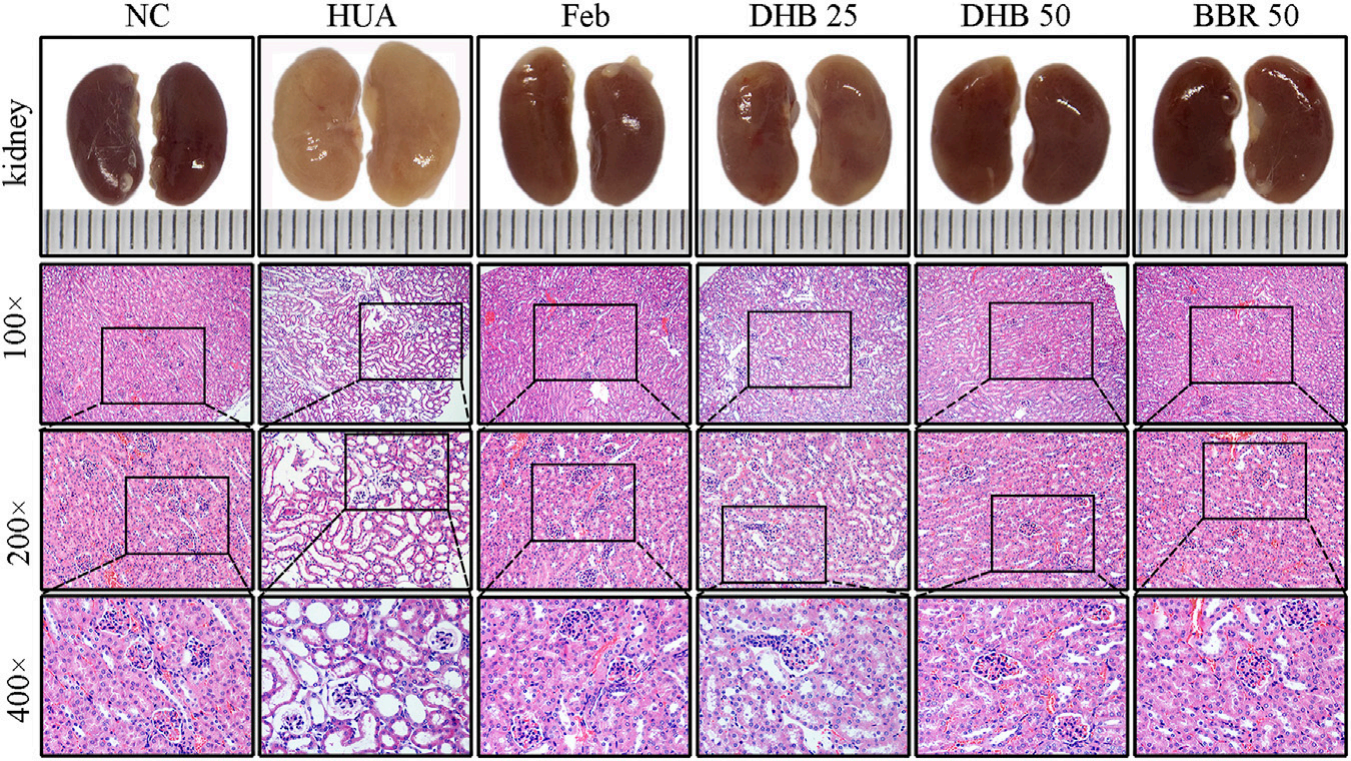
Effect of DHB on kidney general status and histopathological changes in PO/HX-induced hyperuricemic mice. Gross appearance of the kidneys from all six groups were photographed, and sections were subjected to H&E staining; these representative photographs were taken at a magnification of ×100, ×200, ×400, respectively.

The outer appearance and histopathological alterations of kidneys are depicted in Figure 3. In contrast to the kidneys of normal control mice, which had a regular reddish color, the renal tissues in HUA groups were pale and irregularly shaped. However, those changes were ameliorated in a dose-dependent manner by DHB treatment, which potentially contributed to the nephroprotective effect of DHB.

Besides, the renoprotective effect was further verified by histopathological analysis. The mice in the control group showed normal histological features, with neatly arranged renal tubular epithelial cells, normal shape and size of glomeruli, and no evidence of inflammation. While PO/HX-induced hyperuricemic mice displayed severe pathological characteristics of hyperuricemia, characterized by inflammatory cell infiltration, tubular ectasia, and formation of hyaline casts. However, pretreatment with BBR and two doses of DHB significantly attenuated the kidney damage in PO/HX-treated mice, especially the mice in 50 mg/kg DHB group showed more obvious improvement, which retained nearly normal histological features. These results indicated that DHB can ameliorate kidney damage and had renoprotective effect in HUA mice. BBR and Feb showed similar protective effects against pathological alterations.

### Effect of DHB Treatment on XOD and ADA Activity, XOD mRNA and Protein Expression

**FIGURE 4:**
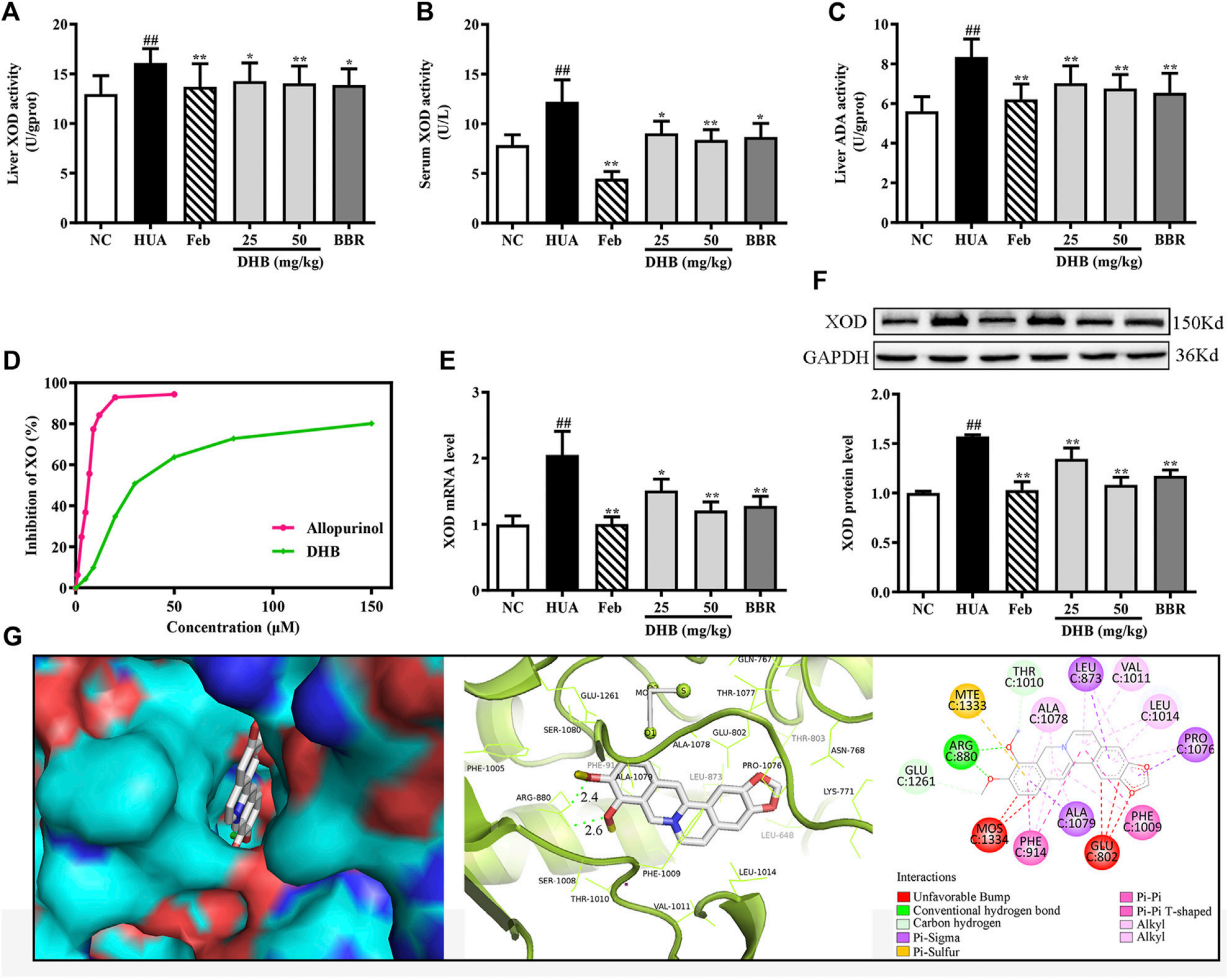
Effect of DHB on XOD *in vitro* and *in vivo*. **(A)** Liver XOD activity, **(B)** serum XOD activity, **(C)** liver ADA activity, **(D)** *in vitro* XOD inhibition by DHB, **(E)** relative mRNA levels of hepatic XOD by RT-qPCR after normalized to β-actin, **(F)** relative protein levels of hepatic XOD by Western blot assay, and GAPDH was used as the control. Bar chart showed quantitative evaluation of XOD bands by optical density. **(G)** Molecular docking modeling of DHB with XOD. The results are expressed as mean ± SD (n = 10). ^*#*^ *p* < 0.05, ^*##*^ *p* < 0.01, compared with NC group; ^*^ *p* < 0.05, ** *p* < 0.01, compared with HUA group.

To evaluate the inhibitory effect of DHB on UA production in mice, hepatic and serum XOD activities, hepatic ADA level, hepatic XOD mRNA and protein expression were determined. As shown in Figures 4A–C, after PO/HX-treatment for 7 consecutive days, hepatic and serum XOD levels were 55.65% and 24.18% higher than those of the normal mice, respectively (all *p* < 0.01). Whereas DHB at 25 and 50 mg/kg and BBR at 50 mg/kg remarkably and dose-dependently attenuated the elevated XOD activities in mice liver and serum. Moreover, Feb also exhibited similar effect and decreased serum and hepatic XOD activities approximately by 63.73% and 14.95% as compared to the HUA group, respectively. In PO/HX-induced hyperuricemic mice, the level of ADA was obviously enhanced by 48.88% (*p* < 0.01) compared with the NC group. However, DHB administration resulted in a remarkable reduction in ADA activity by 15.86% (25 mg/kg, *p* < 0.01) and 19.02% (50 mg/kg, *p* < 0.01), respectively. Besides, a greater decrease in the hepatic ADA level was observed for Feb and BBR group, with the inhibitory rate of 27.23% (*p* < 0.01) and 21.65% (*p* < 0.01), respectively.

The result in Figures 4E,F indicated that in the HUA group, the gene and protein expression levels of XOD were observably up-regulated by PO/HX. However, treatment with DHB noticeably down-regulated the mRNA and protein expression of XOD, and high-dose DHB (50 mg/kg) possessed more obvious modulatory effect. While intervention with allopurinol caused a significant reduction in the hepatic XOD mRNA and protein expression.

### *In Vitro* XOD Enzymatic Activity Inhibition Assay

To evaluate the inhibitory activity of DHB against XOD, an *in vitro* XOD inhibitory assay was performed. As shown in Figure 4D, DHB showed XOD enzyme inhibitory effect in a concentration-dependent manner. DHB significantly inhibited XOD activity with an IC_50_ value of 34.37 ± 2.84 μM, which was higher than allopurinol’s IC_50_ value of 5.80 ± 0.69 μM. These data indicated that DHB had inhibitory effect on XOD.

### Molecular Docking Study

The molecular docking results of DHB binding with XOD are presented in Figure 4G. This finding indicated that DHB could enter into the hydrophobic pocket and interact with the active site of XOD with a binding energy of -9.5 kcal/mol. The oxygen atoms in 9,10-dimethoxy of DHB bonded to the ARG880 of XOD by two hydrogens bonds with bond lengths of 2.4 and 2.6 Å, respectively. Also, it bonded with PHE914 and PHE1009 through Pi–Pi interactions and ALA1079, PRO1076, and LEU873 through Pi-Sigma interactions.

### Effect of DHB Treatment on GLUT9 and URAT1

**FIGURE 5:**
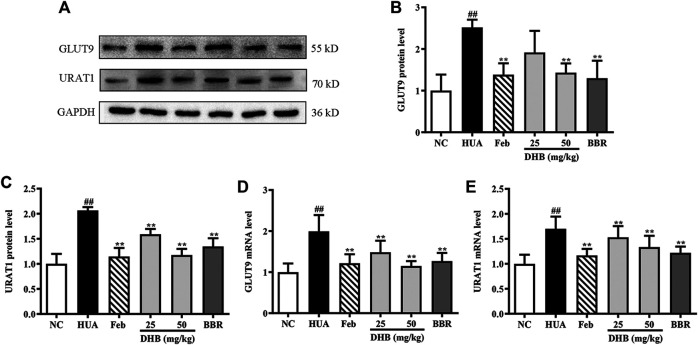
Effect of DHB on renal gene and protein expression of URAT1 and GLUT9 in PO/HX induced hyperuricemic mice. **(A)** Representative Western blot bands, **(B)** protein expression of GLUT9, **(C)** protein expression of URAT1, **(D)** mRNA expression of GLUT9, **(E)** mRNA expression of URAT1. The protein and mRNA expressions of GLUT9 and URAT1 were detected by Western blot and RT-qPCR in the renal tissues, respectively. The results are expressed as mean ± SD (n = 3–5). ^*#*^ *p* < 0.05, ^*##*^ *p* < 0.01, compared with NC group; ^*^ *p* < 0.05, ** *p* < 0.01, compared with HUA group.

Figure 5 depicts the influence of DHB on mRNA and protein expression of GLUT9 and URAT1 in PO/HX-induced HUA mice. PO/HX challenge remarkably elevated kidney URAT1 and GLUT9 gene expression levels (*p* < 0.01) compared with the NC group. However, the increased URAT1 and GLUT9 mRNA levels in HUA mice were markedly lowered by treatment with DHB and BBR. In addition, PO combined with HX administration resulted in prominent augmentation on the protein expression levels of URAT1 and GLUT9. Nevertheless, DHB and BBR at 50 mg/kg effectively restored HUA-induced over-expression of URAT1 and GLUT9 protein. However, DHB at 25 mg/kg had no significant influence on GLUT9 (*p* > 0.05 vs*.* HUA).

### Effect of DHB Treatment on Kidney Inflammatory Cytokines

**FIGURE 6:**
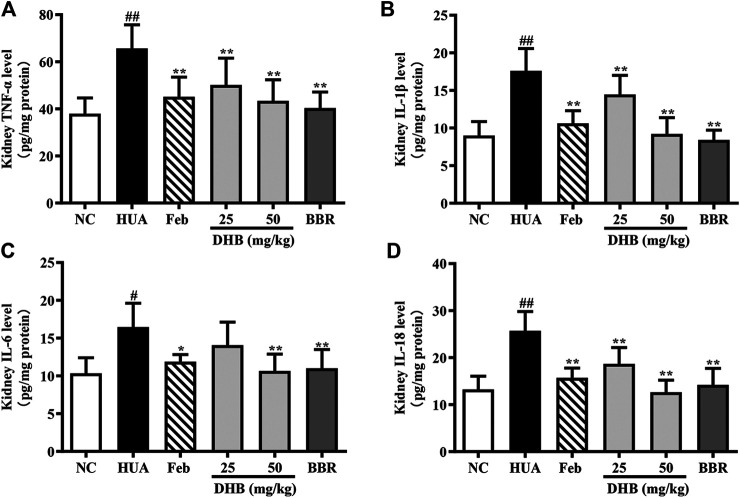
Effect of DHB on kidney inflammation cytokines levels in PO/HX-induced hyperuricemic mice by ELISA. **(A)** TNF-α (B) IL-1β, **(C)** IL-6, **(D)** IL-18. The results are expressed as mean ± SD (n = 3–5). ^*#*^ *p* < 0.05, ^*##*^ *p* < 0.01, compared with NC group; ^*^ *p* < 0.05, ** *p* < 0.01, compared with HUA group.

To assess the renoprotective action of DHB in HUA mice, the levels of inflammatory cytokines TNF-α, IL-1β, IL-6 and IL-18 in renal tissue were determined and the results are depicted in Figure 6. As expected, the levels of TNF-α, IL-1β, IL-6 and IL-18 in PO/HX-induced hyperuricemic mice notably increased by 72.98%, 95.65%, 59.21%, and 93.98%, respectively. Furthermore, 25 and 50 mg/kg DHB remarkably and dose-dependently attenuated the increased inflammatory cytokines production relative to the model group. Allopurinol and BBR also led to a substantial reduction in these four parameters. These findings suggested that DHB could effectively ameliorate PO/HX-induced renal inflammation.

### Effect of DHB Treatment on NLRP3 Inflammasome Activation

**FIGURE 7:**
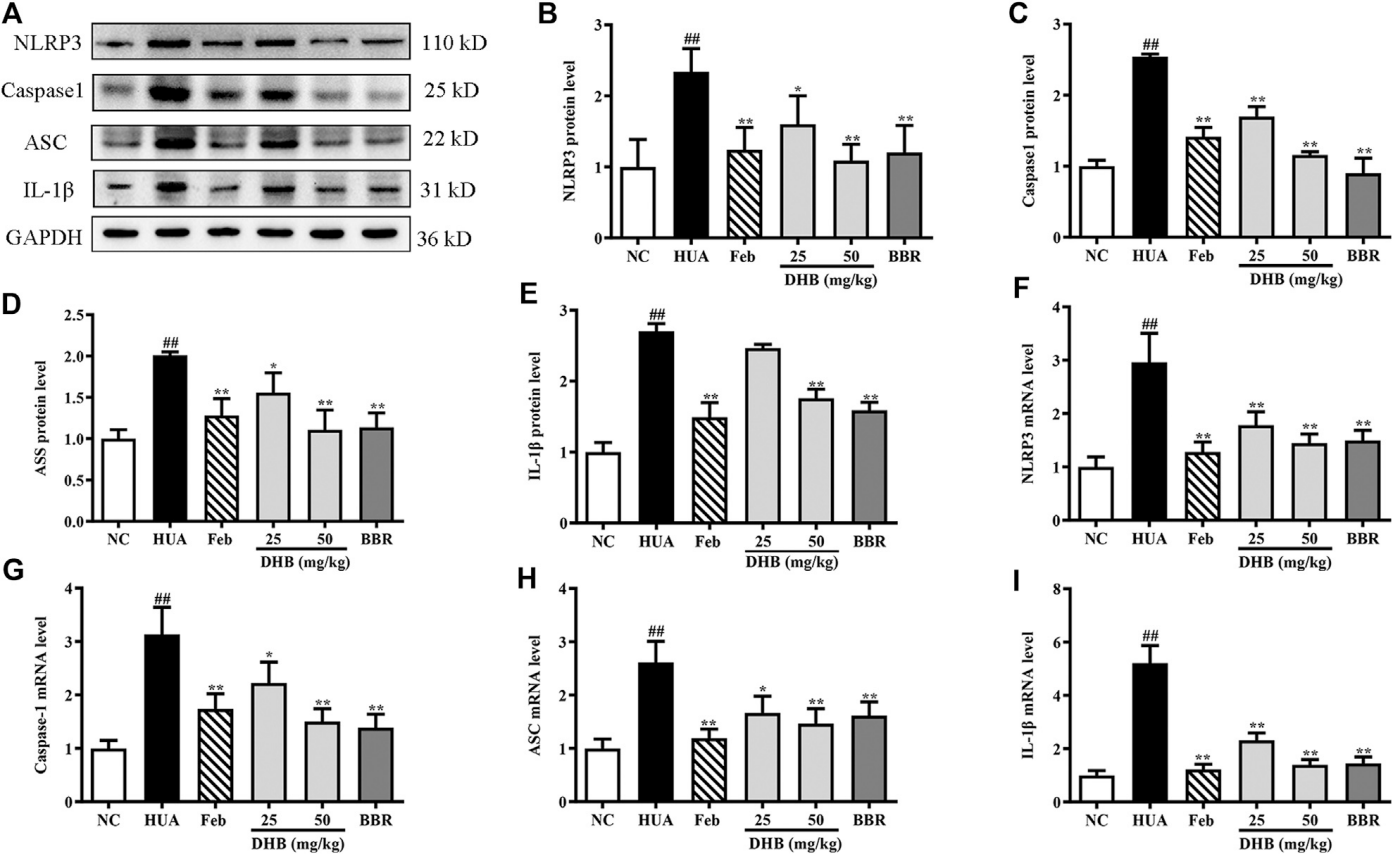
Effect of DHB on NLRP3 inflammasome signaling in *p*O/HX-induced hyperuricemic mice. **(A)** Representative Western blot bands; The protein expression of **(B)** NLRP3, (C) caspase-1, **(D)** ASC, **(E)** IL-1β; the gene expression of **(F)** NLRP3, **(G)** caspase-1, **(H)** ASC, **(I)** IL-1β; Protein levels were determined by Western blotting and the band optical densities were quantitated by Image J software. The renal protein levels were normalized to GAPDH. Relative gene levels were determined by RT-qPCR. The results are expressed as mean ± SD (n = 3–5). ^*#*^ *p* < 0.05, ^*##*^ *p* < 0.01, compared with NC group; ^*^ *p* < 0.05, ** *p* < 0.01, compared with HUA group.

Real-time PCR and Western blotting were performed to explore whether the mechanism of DHB on alleviating inflammation was related to the NLRP3 inflammasome. The result in Figure 7 indicates that PO/HX challenge triggered a remarkable augmentation on the mRNA expression of NLRP3 (increased by 196.14%, *p* < 0.01), ASC (increased by 161.09%, *p* < 0.01), caspase-1 (increased by 213.33%, *p* < 0.01), and IL-1β (increased by 421.09%, *p* < 0.01), respectively. In contrast, administration with DHB or allopurinol noticeably down-regulated the mRNA expression of NLRP3, ASC, caspase-1, and IL-1β. After BBR treatment at 50 mg/kg, the gene expression of NLRP3, ASC, caspase-1, and IL-1β was 49.56, 38.21, 55.70, and 72.13% (all *p* < 0.01) lowered than that of the HUA group, respectively. In parallel with the previous mRNA results, DHB treatment significantly ameliorated PO-induced over-expression of NLRP3, ASC, caspase-1 and IL-1β protein. Furthermore, compared to the HUA group, allopurinol at 5 mg/kg also remarkably suppressed renal NLRP3, ASC, caspase-1, and IL-1β protein expression in hyperuricemic mice. And similar inhibitory actions were also observed in BBR (50 mg/kg).

## Discussion

Currently, numerous mouse models have been successfully established to investigate the molecular mechanisms of hyperuricemia. These models can be divided into four categories based on different ways to elevate UA levels (Pang et al., 2017; Chau et al., 2019; Lu et al., 2019): 1) direct supplement of UA, UA precursors, or high-purine food, such as xanthine, hypoxanthine, fructose, or yeast; 2) inhibition of UA excretion, such as ethambutol or nicotinic acid; 3) blockage of the activity of uricase, such as potassium oxonate; 4) genetically modified models, such as Glut9-knockout, Abcg2-knockout and Urat1-knockout mice models. Uricase is an enzyme that oxidizes UA to the water-soluble allantoin. PO is known as a selectively competitive inhibitor of uricase, which can dramatically elevate the concentration of UA. Therefore, it has been frequently employed to establish hyperuricemia animal models. Hypoxanthine is converted to xanthine and further catalyzed to UA by XOD. Hence, over-supplementation of hypoxanthine can also directly increase the levels of UA. To establish a relatively stable and durable hyperuricemia model, many studies have combined administration of two or three model drugs (Yong et al., 2016; Yong et al., 2017). Consistent with previous reports, we found that UA levels were observably increased after 7 days of HX and PO co-administration, which indicated the successful construction of HUA model.

Xanthine oxidoreductase (XOR), a key rate-limiting enzyme highly expressed in the liver, plays a vital role in catalyzing the last two steps of purine catabolism. XOR consists of two interconvertible forms, xanthine dehydrogenase (XDH) and xanthine oxidase (XOD). XDH is the primary gene product of XOR, and XOD is formed through the post-translational modification of XDH (Vorbach et al., 2003). Among these two forms, XOD is crucial in UA production. High XOD levels lead to the overproduction of UA. Hence, inhibition of XOD has been widely accepted as a significant strategy for the treatment of HUA. Allopurinol and febuxostat are two major drugs for treating HUA as classic XOD inhibitors. However, some undesirable adverse effects compromise their therapy usage. Furthermore, structural analysis has indicated that XOD consists of three domains, and molybdopterin is the largest one that acts as the critical active site in XOD (Okamoto et al., 2013). The molybdenum cofactor is enclosed by diverse amino acid residues: Phe649, Phe914, Phe1009, Asn768, Val1011, Glu802, Ser876, Lys771, Leu873, Leu1014, Arg880, Thr1010, Glu1261, etc., among which Glu802, Arg880, and Glu1261 are the most important residues for stimulating the molybdopterin active center. These amino acid residues form hydrogen bond with XOD inhibitors to inhibit the enzymatic activity of XOD (Li et al., 2018). DHB is a natural hydrogenated derivative of BBR, and has been identified from the PC ethyl acetate fractions for the first time in our previous work (Tan et al., 2019). Besides, Feng et al. have reported that gut bacteria in feces could transform BBR into DHB, which displayed a 5-fold higher intestinal absorption rate than BBR (Feng et al., 2015). Interestingly, DHB displayed more obvious *in vivo* efficacy in the treatment of experimental diabetes (Turner et al., 2008), hyperlipidemia (Wei et al., 2014; Liu et al., 2015), and atherosclerosis (Chen et al., 2014). Hence, DHB has the potential to be more valuable and promising than BBR for the development of new agents. However, no information is available on the mechanism underlying the potential anti-hyperuricemia and renoprotective effects of DHB, nor their relationships as compared to BBR. Previous studies have indicated that alkaloids have potential anti-hyperuricemia effect (Liu et al., 2014; Wang et al., 2015). And our findings are in parallel with previous reports that DHB administration substantially decreased SUA level, serum and liver XOD activities, XOD mRNA and protein expression in HUA mice. Studies *in vitro* showed that DHB possessed remarkable XOD suppressive effect in a dose-dependent manner. Furthermore, the molecular docking simulation further supported the excellent XOD inhibitory activity of DHB. Our finding indicated that DHB exhibited promising hypouricemic effect in hyperuricemic mice and the underlying mechanism may be partly due to XOD inhibition.

Furthermore, under-excretion of renal UA is responsible for the development of hyperuricemia (Zhang et al., 2018). Kidney, a main regulator of SUA levels, plays a prominent role in maintaining blood urate homeostasis via interaction between the secretion and reabsorption of urate in renal tubule. In humans, about 90% of filtered urate is reabsorbed into blood by urate-transport-related proteins located along the renal proximal tubule (Xu et al., 2020). URAT1 and GLUT9 are two most important and widely studied molecular targets related to the UA reabsorption. Studies have shown that dysregulation of URAT1 and GLUT9 alters urate handling, which should be responsible for the occurrence of hyperuricemia. To further investigate the possible mechanism underlying the hypouricemic action of DHB, the gene and protein expression levels of URAT1 and GLUT9 were also analysed. In this study, DHB treatment effectively in restored the HUA-caused over-expression of URAT1 and GLUT9 at both mRNA and protein levels. Hence, the hypouricemic effect of DHB may be partly attributed to the improvement of renal urate excretion.

The renoprotective action of DHB in PO/HX-induced HUA mice was also studied. Serum BUN and creatinine are the two most widely used indexes to assess renal function. Generally, PO/HX challenge could lead to serious renal damages along with elevated serum BUN and creatinine levels, which marked a decreased clearance of urea and creatinine (Zhang et al., 2015). Besides, it is widely accepted that the alterations in body weight, organ weight, and organ index are sensitive signs for evaluation of the toxic effects of chemicals for inner organs (Dutta and Sahu, 2013). In this study, combined supplementation of PO and HX once daily for 7 consecutive days remarkably increased serum BUN and creatinine levels as compared to the NC group, indicative of the occurrence of renal dysfunction. Conversely, DHB treatment significantly reversed the elevated BUN and creatinine levels as compared to the HUA group. These results showed that in contrast to the noticeable nephrotoxicity of model drugs employed, DHB treatment could effectively lower the kidney weight and kidney coefficients. In sum, our findings suggested that DHB was beneficial to inner organs including renal function. Furthermore, these properties were further validated by histopathological analysis. The results showed that PO/HX-induced kidney impairment was notably ameliorated, and a remarkable reduction of the amount of inflammatory cells and improvement in tubular ectasia and formation of hyaline casts were observed after DHB treatment. The results were in line with the previous studies (Hongyan et al., 2016). Together, DHB at 25 mg/kg showed promising anti-hyperuricemic and nephroprotective effects in PO/HX-induced hyperuricemic mice. If an extrapolation of the above results is to be made to humans, then it may be said that, the minimum anticipated biological-effect level (MABEL) 25 mg/kg (approximately equal to ∼121.5  mg/60  kg body weight for adult dose in terms of body surface area dose translation), might be deemed as a possible starting dose for phase I clinical trial, regarding the above exposure–response data. However, standard preclinical safety evaluation as well as pharmacokinetic/pharmacodynamics model should be carried out prior to further potential clinical trial.

Many lines of evidence has demonstrated that inflammation is a typical pathologic feature of hyperuricemia, which acts a pivotal part in the progression of hyperuricemia (Zhang et al., 2015). In PO/HX-induced hyperuricemic mice, the expression levels of pro-inflammatory cytokines (IL-1β, TNF-α, IL-6 and IL-18) are aberrantly increased, which lead to an inflammatory reaction, and further cause kidney impairment (Zhou et al., 2012). TNF-α, a pro-inflammatory cytokine produced by mononuclear cells and macrophages, is closely related to the severity of MSU-induced gout. Indeed, TNF-α directly promotes the expression of IL-1β protein and pro-IL-1β mRNA transcription in a mouse model of gout, and blocks TNF-α resulting in a dramatic amelioration of inflammation (Amaral et al., 2016). IL-1β, an indicator of gout inflammation, plays a key role in MSU-induced initiation and progression of acute gout flares (Dalbeth et al., 2016). IL-6 is also an important proinflammatory cytokine that exerts an enormous impact on gout pathogenesis. Guerne et al. have shown that synoviocytes and monocytes exposed to UA significantly increased the production of IL-6 (Guerne et al., 1989). Our previous study has found that DHB displayed anti-inflammatory effects, including substantially decreasing the production and mRNA expressions of IL-6, IL-1β, and TNF-α in three classic acute inflammatory murine models and LPS- stimulated RAW 264.7 cells (Li et al., 2019; Tan et al., 2019). Our study suggested that the levels of IL-6, IL-1β, TNF-α and IL-18 in the kidney were markedly reduced by DHB, which indicated the anti-inflammatory efficacy of DHB in PO/HX-induced HUA mice.

As mentioned above, the release of pro-inflammatory cytokines is the key of kidney inflammation, and NLRP3 inflammatory pathway plays a crucial role in regulating inflammation (Wu et al., 2017). The NLRP3 inflammasome is an intracellular multiprotein complex composed of a receptor NLRP3 (NACHT, LRR, and PYD domain-containing protein 3, cytosolic sensor protein), an adaptor ASC (an apoptosis-associated speck-like protein containing a caspase recruitment domain), and an effector caspase-1 (cysteinyl aspartate specific proteinase-1). An increasing amount of evidence indicates that high urate level directly activates the NLRP3 inflammasome and subsequently promotes IL-1β maturation and secretion, contributing to kidney inflammation and renal dysfunction (Liu et al., 2019). Our results showed that NLRP3 inflammasome was activated in hyperuricemic mice, as evidenced by up-regulating renal mRNA and protein expression of NLRP3, caspase-1, ACS, and IL-1β. These increases were remarkably suppressed by DHB, BBR and febuxostat.

## Conclusion

**FIGURE 8:**
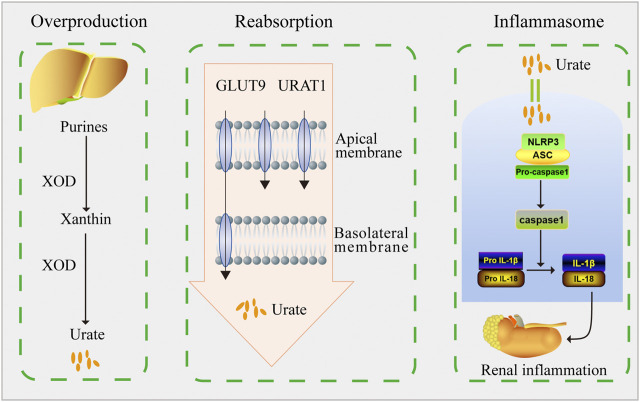
Schematic diagram of this study. DHB exhibited dual anti-hyperuricemic effects via inhibiting hepatic XOD activity as well as down-regulating renal URAT1 and GLUT9 expression. Moreover, DHB possessed renoprotective effect by inhibiting NLRP3 inflammasome activation.

In conclusion, our findings demonstrated that DHB exerted significant hypouricemic and nephroprotective effects in PO/HX-induced HUA mice. DHB displayed the XOD-inhibitory ability *in vitro* and *in vivo*. Furthermore, DHB administration resulted in prominent inhibition on renal URAT1 and GLUT9 levels. Therefore, DHB exhibited dual anti-hyperuricemic effects via inhibiting hepatic XOD to reduce urate formation as well as down-regulating renal URAT1 and GLUT9 to enhance urate excretion. Moreover, DHB was also found to possess renoprotective effect, at least in part by inhibiting NLRP3 inflammasome activation to mitigate kidney inflammation and dysfunction (Figure 8). Hence, our findings suggested that DHB had the potential to be developed into a safe and effective therapeutic agent for the prevention and treatment of hyperuricemia.

## Data Availability

The raw data supporting the conclusions of this article will be made available by the authors, without undue reservation.
